# Supplementary material for: Head movement dynamics in dystonia: a multi-centre retrospective study using visual perceptive deep learning
Source: NPJ Digit Med. 2024 Jun 18;7:160. doi: 10.1038/s41746-024-01140-6 (PMC11189529; doi:10.1038/s41746-024-01140-6)
Supplement: Supplementary file 1 — Supplementary Information [file 41746_2024_1140_MOESM1_ESM.pdf]

Supplementary Information

Robert Peach[1,2,*,†], Maximilian Friedrich[1,3,4,*], Lara Fronemann[1], Muthuraman Muthuraman[1], Sebastian R. Schreglmann[1], Daniel Zeller[1], Christoph Schrader[5], Joachim K. Krauss[6], Alfons Schnitzler[7], Matthias Wittstock[8], Ann-Kristin Helmers[9], Steffen Paschen[10], Andrea Kühn[11], Inger Marie Skogseid[12], Wilhelm Eisner[13], Joerg Mueller[14], Cordula Matthies[15], Martin Reich[1], Jens Volkmann[1,‡], Chi Wang Ip[1,‡,†]

[1]Department of Neurology, University Hospital Würzburg, Würzburg, 97070, Germany

[2]Department of Brain Sciences, Imperial College London, London, United Kingdom,

[3]Center for Brain Circuit Therapeutics, Brigham & Women's Hospital, Boston, USA,

[4]Harvard Medical School, Boston, USA,

[5]Department of Neurology and Clinical Neurophysiology, Hannover Medical School, Hannover, Germany,

[6] Department of Neurosurgery, Hannover Medical School, Hannover, Germany.

[7] Institute of Clinical Neuroscience and Medical Psychology, Heinrich Heine University Düsseldorf, Düsseldorf, Germany.

[8] Department of Neurology, University Hospital Rostock, Rostock, Germany.

[9] Department of Neurology, UKSH, Kiel Campus Christian-Albrechts-University, Kiel, Germany.

[10] Department of Neurology, Christian-Albrechts-University, Kiel, Germany.

[11] Department of Neurology, Movement Disorder and Neuromodulation Unit, Charité - Universitätsmedizin Berlin, Germany.

[12] Movement Disorders Unit, Department of Neurology, Oslo University Hospital, Rikshospitalet, Oslo, Norway.

[13] Department of Neurology, Innsbruck Medical University, 6020 Innsbruck, Austria.

[14] Klinik für Neurologie mit Stroke Unit, Vivantes Klinikum Spandau, Berlin, Germany.

[15]Department of Neurosurgery, University Hospital Würzburg, Würzburg, 97070, Germany

[*] Joint first authors

[‡] Joint last authors

[†]To whom correspondence should be addressed; E-mail: peach_r@ukw.de; ip_c@ukw.de

Supplementary Discussion

Evidence before this study

Clinical assessment of dystonia, a neurological movement disorder, has traditionally relied on rating scales that aim to simplify complex phenomenology into lower-dimensional rating items. However, these score-based assessments have significant clinimetric limitations and do not fully capture the rich spatiotemporal dynamics of dystonic phenomena, which are crucial for clinical judgment and pathophysiological understanding. In contrast, recent investigations in animal models of dystonia have already demonstrated the utility and relevance of quantitative methods for phenotyping, which gradually supersedes previous observer-dependent behavioural analyses. Taken together, this has led to a need for more objective and detailed clinical evaluation methods of dystonia.

We performed a PubMed search up to July 2023 combining the terms "dystonia" AND ("deep learning" OR "machine learning" or "computer vision" OR "vision-based" OR "video-based") AND ("angle" OR "kinematic" OR "rating" OR "scoring" OR "movement analysis") including abstracts

in English or German. The search yielded three studies that validated vision-based frameworks for automating the assessment of cervical dystonia severity compared to clinician-annotated ratings. Two of these studies focused on deriving head angle deviations from specialised camera setups, while the third study utilised computer vision in a retrospective video dataset recorded using conventional equipment. These studies reported fair to moderately strong correlations between vision-based head angle measurements and clinical scores. Additionally, three studies investigated computer vision for assessing head tremor in the context of cervical dystonia: one case report demonstrated the clinical validity of computer vision-derived head angle and head tremor metrics, while a retrospective cross-sectional study reported moderate agreement of computer vision-derived metrics and scores, and another retrospective study used computer vision to analyze the interplay of static and dynamic components of cervical dystonia. Two additional studies used computer vision-based kinematics to quantify dystonia-like phenomena in rodent models of monogenetic dystonia, demonstrating utility in both phenotype and genotype predictions.

However, most of the clinical studies were limited to static task conditions, where patients attempted to hold a neutral position of the head, thus not providing a naturalistic account of dystonia. Moreover, beyond head angular deviations and oscillation metrics, no study explored a broader kinematic feature space that reflects the true spatiotemporal complexity of dystonic movements. Additionally, the studies assessed patients at single time points without considering different therapy conditions, particularly the effects of deep brain stimulation, which is a highly effective intervention targeting brain circuits. Nor did they compare dystonia sub-types, such as cervical and generalised dystonia.

Added value of this study

In this study, we present a comprehensive visual perceptive deep learning framework that addresses the gaps in current dystonia assessments. We use this framework to retrospectively analyse a unique dataset from three multi-centric, studies encompassing video examinations of patients along the dystonic severity continuum, including different deep brain stimulation states. Our framework goes beyond the automation of suboptimal symptom severity assessments by reverse engineering a set of clinically inspired kinematic features. The resulting high dimensional, yet intuitively interpretable kinematic feature space enabled us to explore disease states and effects of brain circuit therapies in a level of detail comparable to experimental neuroscientific investigations. Through a data-driven approach, we have identified a consistent set of only four dynamic parameters that encode dystonia severity, subtype, and the efficacy of brain circuit interventions. Notably, these features are independent of static head angle deviations, which play a central role in dystonia severity scores, pointing to the involvement of partially distinct neurobiological processes not captured by these scores. Our findings align with emerging concepts of symptom-specific brain circuits and findings in rodent models of dystonia, thereby exemplifying the visual perceptive framework's potential to augment clinical management and bridge translational gaps in movement disorders research. By providing a more comprehensive and precise assessment of the disorder, our study offers valuable insights for improved treatment strategies and further understanding of dystonia's complex neurobiology.

Implications of all the available evidence

The available evidence collectively underscores the limitations of traditional rating scales in capturing the informative spatiotemporal dynamics of dystonic movements, emphasizing the need for more objective and granular evaluation methods. In line with recent animal studies using computer vision for dystonia quantification, recent clinical studies have shown the potential of computer vision-based frameworks in automating cervical dystonia severity assessment and capturing head tremor metrics. However, their underlying study designs may inadvertently reinforce limitations associated with the clinical scoring process.

In this study, we introduce a comprehensive visual perceptive deep learning framework that serves as a powerful platform to augment clinical judgement and generate valuable pathophysiological insights by extracting a set of clinically inspired, interpretable kinematic features. Our findings have implications beyond dystonia, showcasing the utility of visual perceptive frameworks in enhancing clinical management and fostering integration with advanced neuroimaging and neurotechnological methods. This study opens doors for future translational research to explore the broader application

of computer vision and deep learning techniques to derive kinematic signatures of movement disorders across species and experimental conditions, promising more precise and personalised assessments that can significantly improve therapeutic strategies and patient outcomes.

Model variable interpretation

We remind the reader that Mediapipe was used to detect a face mesh, enabling us to determine the head angles along three axes of motion: torticollis (rotation), laterocollis (tilt), and antero-retrocollis (forward and backward). The custom trained CNN was used to predict the movement state, e.g., rotation left or tilt right. Predictions were made for each frame of the video, after which we engineered features that capture the dynamics of the patient. Below we provide more details on the interpretation of the derived features from the two deep learning tools.

Head angles (derived from Mediapipe face-mesh tracking):

- Angle torticollis: Head-angle deviation from face-forward in yaw axis when a patient is sitting in a neutral position. Positive angle = right, Negative angle = left.
- Angle laterocollis: Head-angle deviation from face-forward in tilt axis when a patient is sitting in a neutral position. Positive angle = right tilt, Negative angle = left tilt.
- Angle antero/retrocollis: Head-angle deviation from face-forward in antero/retrocollis axis when a patient is sitting in a neutral position. Positive angle = anterocollis, Negative angle = retrocollis.

Correlation features (derived from custom CNN movement state prediction):

- Correlation movement mean: Mean correlation coefficient between all predicted movement states.
- Correlation mean face forward: Mean correlation coefficient of each movement state to face-forward movement state.

Head oscillations (derived from head angles, i.e., Mediapipe):

- Oscillation amplitude: The amplitude of the largest peak in a Fourier transform of the angles. For each axis respectively.
- Oscillation frequency: The frequency of the largest peak in a Fourier transform of the angles. For each axis respectively.

Symmetry features (derived from custom CNN movement state prediction):

- Symmetry rotation: Proportion of time head was oriented in one direction compared to the opposite direction for the rotation states (left or right).
- Symmetry tilt: Proportion of time head was oriented in one direction compared to the opposite direction for the tilt states (left or right).
- Symmetry anteroretrocollis: Proportion of time head was oriented in one direction compared to the opposite direction for the antero/retrocollis states (forward or backward).

Harmonics (derived from head angles, i.e., Mediapipe): The harmonic strengths were determined using the head angles for each axis of motion respectively. The harmonic strength was determined as the distance correlation between the fundamental tremor frequency and its first harmonic (double the fundamental frequency).

Supplementary figures

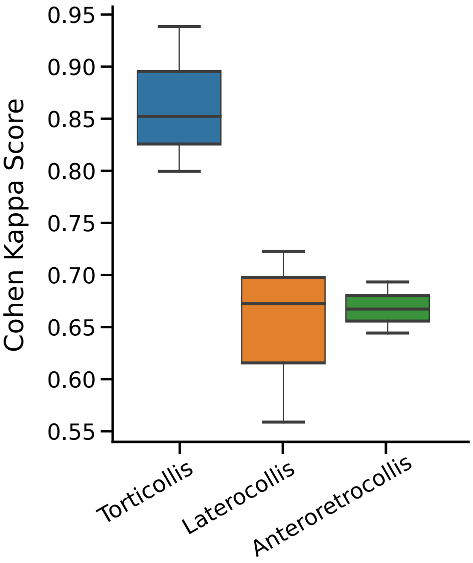

Supplementary Figure 1: **Agreement of clinical raters.** Distribution of Cohen-Kappa-scores by axis (N=86, 3-paired comparisons per axis).

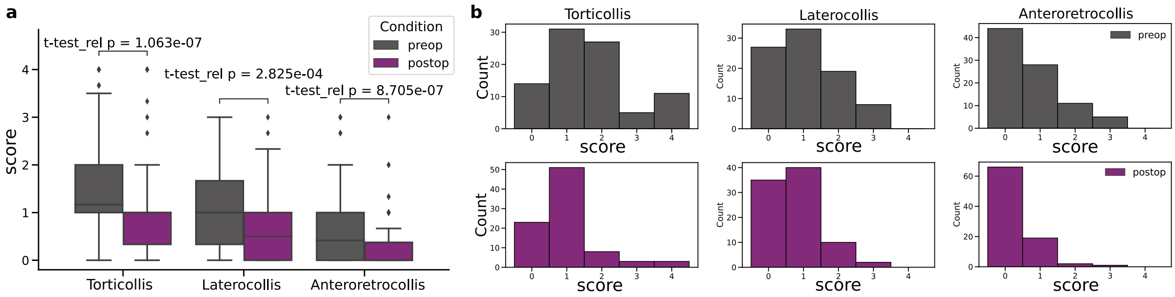

Supplementary Figure 2: **Distribution of pre- and post-operative clinically annotated scores. A** Box plots showing clinically rated patient scores (mean across clinical raters) for pre- (grey) and post- (purple) operation, for each axes of movement. Median and interquartile ranges are displayed in each plot. **B** Pre- (top) and post- (bottom) operative distributions of scores. Maximum score is 4 for torticollis and 3 for laterocollis and anteroretrocollis. N=86.

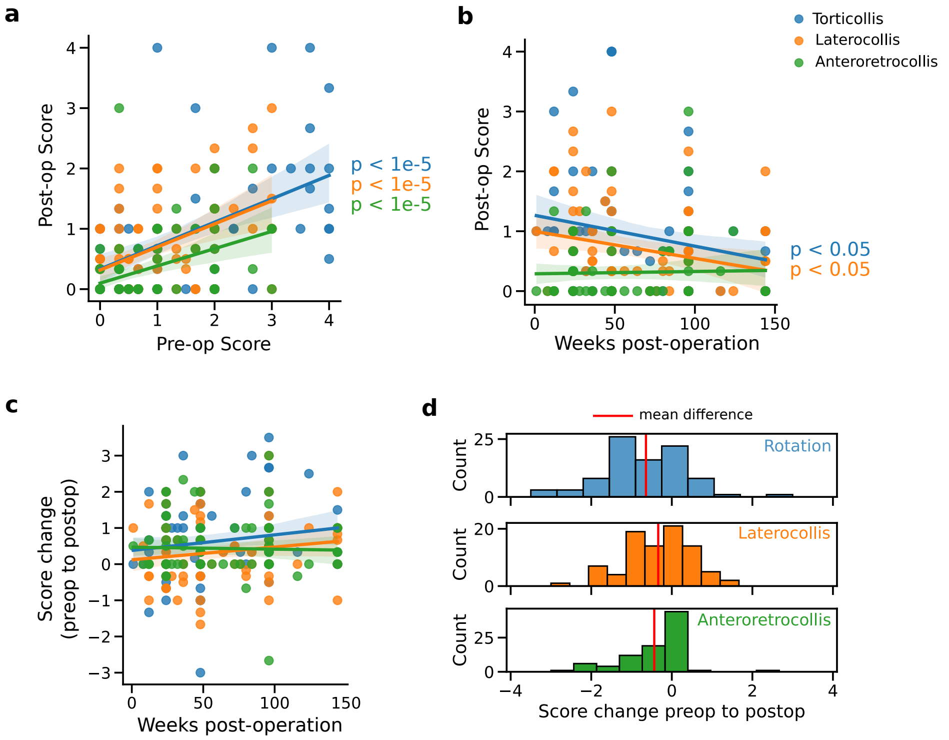

Supplementary Figure 3: **Comparison of pre- and post-operation scores.** **A** Scatter plot showing correlations of pre- and post-operation scores. Fitted linear model. **B** Correlation between date of post-operative assessment and score. **C** A scatter plot of the relative change in clinicallly assigned scores between pre- and post-op (pre-op score subtracted from post-op score). **D** Histograms of relative changes in scores (pre-op score subtracted from post-op score). By colours: torticollis (blue), laterocollis (orange) and anteroretrocollis (green). N=86.

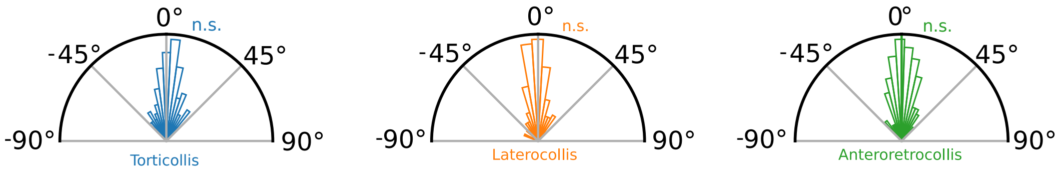

Supplementary Figure 4: **Deviations from face-forward are centred at zero at the group-level.** Polar histograms showing that the average angle does not significantly deviate in any direction. One-sample t-tests were non-significant in each axis of motion (N=86).

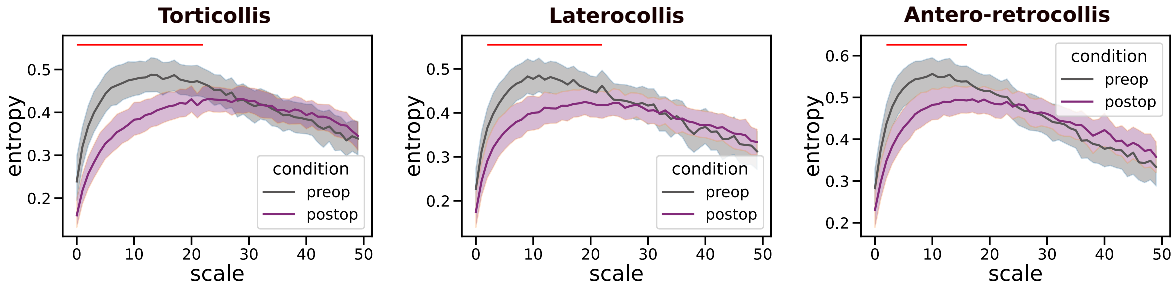

Supplementary Figure 5: **Multiscale entropy reveals only short timescale differences pre- to post-operation.** Multiscale entropy analysis (approximate entropy) is applied to the each head-angle time-series for increasing scales (Python EntropyHub 0.2). Scale (x-axis) is defined in units of video frames (videos were standardised to a sampling rate of 25 frames per second). Maximal entropy is observed earlier for pre-operative recordings relative to post-operation. Red lines indicate scales with significant differences between pre and post-operation (N=86, paired t-test, $p < 0.05$).

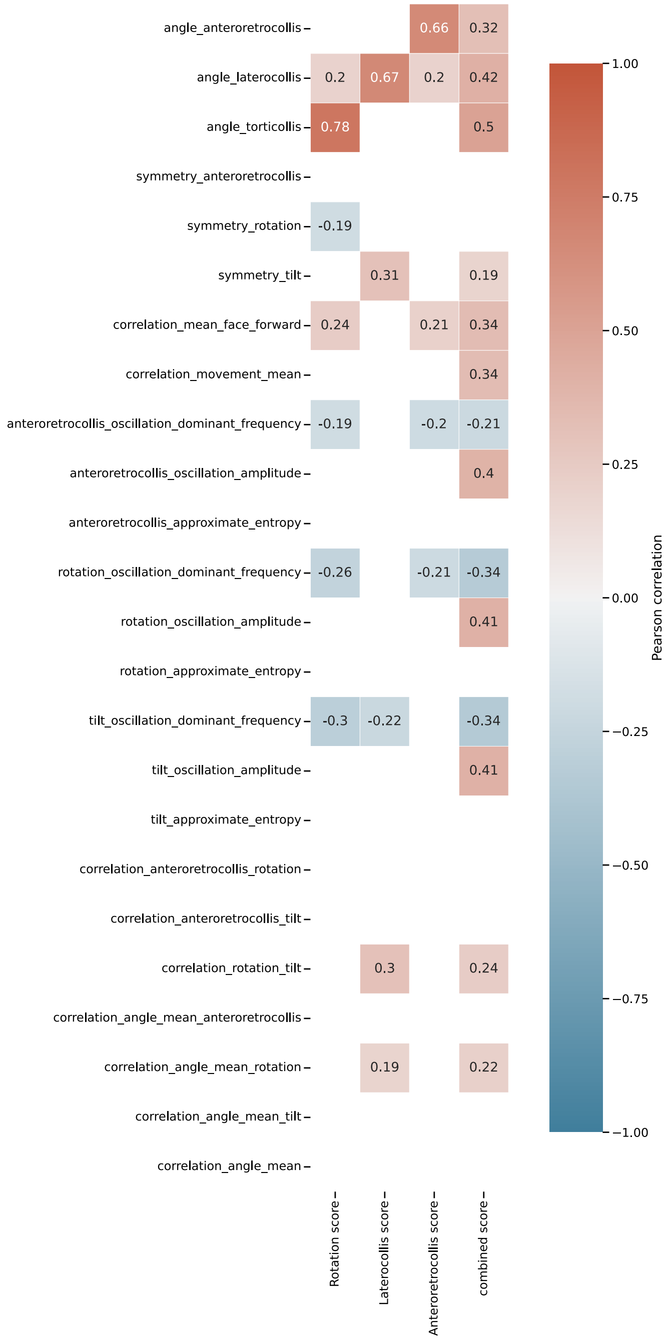

Supplementary Figure 6: **Correlation of kinematic features with annotated scores (cervical dystonia).** Clinically annotated scores (mean across raters) for head-angle deviations from neutral face-forward along each axis are correlated with engineered kinematic features from full videos. A holistic score (taken as the mean clinical rating across the three axes) is also correlated with kinematic features. Only significant (FDR corrected, $p < 0.05$) correlations are shown (N=71).

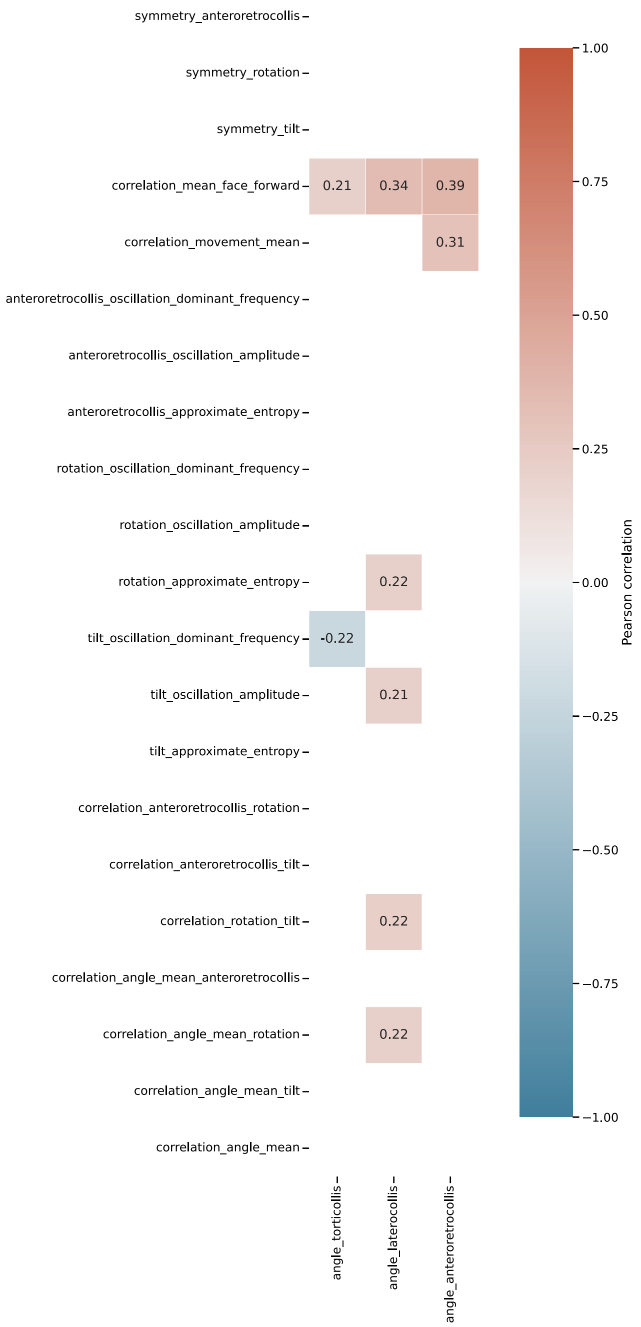

Supplementary Figure 7: **Correlation of kinematic features with face-forward angles (cervical dystonia).** Head-angle deviations during attempted neutral face-forward are correlated with engineered kinematic features from full videos. Only significant (FDR multiple comparisons corrected, $p < 0.05$) correlations are shown (N=71).

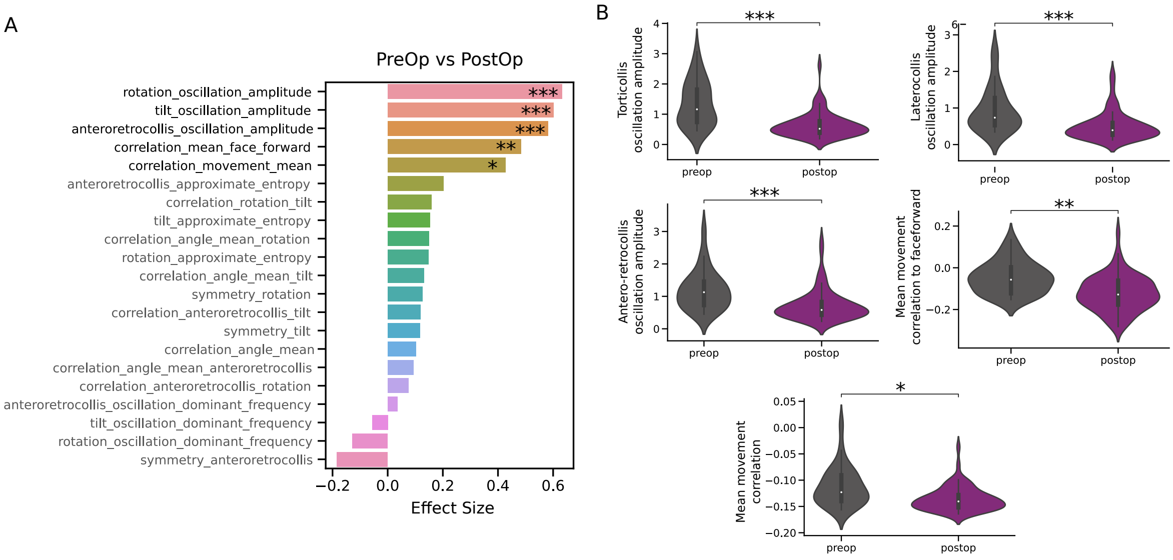

Supplementary Figure 8: **Application of full video kinematics in cohort of generalised dystonia patients. A** Effect size (rank-biserial correlation) of dynamical variables between pre- and post-operation with Wilcoxon tests. **B** Violin plots of variables that are significantly larger pre- (grey) relative to post- (purple) operation. N=30, significance levels: * p< 0.05; ** p< 0.01; *** p< 0.001.

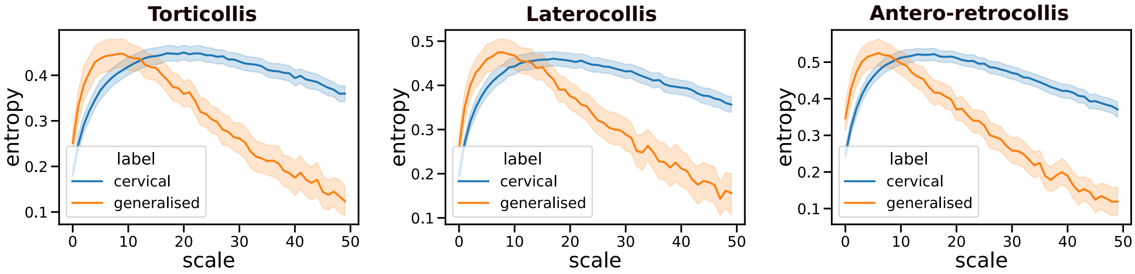

Supplementary Figure 9: **Multiscale entropy reveals differences between pre-operative generalised and cervical patients at all scales.** Multiscale entropy analysis (approximate entropy) is applied to the each head-angle time-series for increasing scales (Python EntropyHub 0.2). Maximal entropy is observed at earlier scales for generalised dystonia patients. N(cervical)=86, N(generalised)=30.

Filming protocol healthy control group TWSTRS

Cluster	Time	Move
	5 sec	Head straight, transition phase
Rotation to the right 30 sec	10 sec	Turn head ¼ range to the right
	10 sec	Turn head ½ range to the right
	10 sec	Turn head full range to the right
	5 sec	Head straight, transition phase
Rotation to the left 30 sec	10 sec	Turn head ¼ range to the left
	10 sec	Turn head ½ range to the left
	10 sec	Turn head full range to the left
	5 sec	Head straight, transition phase
Tilt to the right 30 sec	10 sec	Tilt head ¼ range to the right shoulder
	10 sec	Tilt head ½ range to the right shoulder
	10 sec	Tilt head full range to the right shoulder
	5 sec	Head straight, transition phase
Tilt to the left 30 sec	10 sec	Tilt head ¼ range to the left shoulder
	10 sec	Tilt head ½ range to the left shoulder
	10 sec	Tilt head full range to the left shoulder
	5 sec	Head straight, transition phase
Head to chest 30 sec	10 sec	Head to chest ¼ range
	10 sec	Head to chest ½ range
	10 sec	Head to chest full range
	5 sec	Head straight, transition phase
Head backwards 30 sec	10 sec	Head backwards ¼ range
	10 sec	Head backwards ½ range
	10 sec	Head backwards full range
	5 sec	Head straight, transition phase
Looking straight forward	30 sec	Head straight
	5 sec	Head straight, transition phase
Eye movement 40 sec	10 sec	keep eyes closed
	10 sec	Open eyes
	10 sec	Keep eyes closed
	10 sec	Open eyes
	5 sec	Head straight, transition phase
Shoulder lift right	10 sec	Lift right shoulder
	5 sec	Head straight, transition phase
Shoulder lift left	10 sec	Lift left shoulder
	1-5 sec	Transition phase, end

Supplementary Figure 10: **Healthy controls filming protocol.**
